# Retraction Note to: Peritoneal vaginoplasty by Luohu I and Luohu II technique: a comparative study of the outcomes

**DOI:** 10.1186/s40001-017-0267-8

**Published:** 2017-08-03

**Authors:** Aiwen Le, Zhonghai Wang, Lili Shan, Tianhui Xiao, Rong Zhuo, Guangnan Luo

**Affiliations:** 1 Department of Obstetrics and Gynecology, Affiliated Shenzhen Nanshan People’s Hospital of Guaongdong Medical University, Shenzhen, 518052 Guangdong China; 2 Department of Obstetrics and Gynecology, Affiliated Shenzhen Luohu People’s Hospital of Guaongdong Medical University, Shenzhen, 518052 Guangdong China

## Retraction to: Eur J Med Res (2015) 20:69 DOI 10.1186/s40001-015-0165-x

The authors are retracting this article [1], because of errors in the data that undermine the conclusions of the study. The errors, which were discovered during a follow-up study, resulted from the data-searching methods used and a problem with a computer program.
